# Media actors’ perceptions of their roles in reporting food incidents

**DOI:** 10.1186/1471-2458-14-1305

**Published:** 2014-12-18

**Authors:** Annabelle M Wilson, Julie Henderson, John Coveney, Samantha B Meyer, Trevor Webb, Michael Calnan, Martin Caraher, Sue Lloyd, Dean McCullum, Anthony Elliott, Paul R Ward

**Affiliations:** Discipline of Public Health, Flinders University of South Australia, GPO Box 2100, Adelaide, SA 5001 Australia; School of Nursing and Midwifery, Flinders University of South Australia, GPO Box 2100, Adelaide, SA 5001 Australia; School of Public Health and Health Systems, University of Waterloo, 200 University Avenue West, Waterloo, Ontario N2L 3G1 Canada; Behaviour & Regulatory Analysis, Food Standards Australia and New Zealand, PO Box 7186, Canberra, BC ACT, 2610 Australia; School of Social Policy, Sociology and Social Research, University of Kent, Cornwallis North 19 East, Canterbury, Kent, CT2 7NF UK; Centre for Food Policy, Department of Sociology, School of Arts and Social Sciences, City University, Northampton Square, London, EC1V OHB UK; Food Safety and Nutrition Branch, SA Health, PO Box 287, Rundle Mall, SA 5001 Australia; Hawke Research Institute, University of South Australia, GPO Box 2471, Adelaide, SA 5001 Australia

**Keywords:** Food, Food incident, Media, Public health, Public health professional

## Abstract

**Background:**

Previous research has shown that the media can play a role in shaping consumer perceptions during a public health crisis. In order for public health professionals to communicate well-informed health information to the media, it is important that they understand how media view their role in transmitting public health information to consumers and decide what information to present. This paper reports the perceptions of media actors from three countries about their role in reporting information during a food incident. This information is used to present ideas and suggestions for public health professionals working with media during food incidents.

**Methods:**

Thirty three semi-structured interviews with media actors from Australia, New Zealand and the United Kingdom were conducted and analysed thematically. Media actors were recruited via purposive sampling using a sampling strategy, from a variety of formats including newspaper, television, radio and online.

**Results:**

Media actors said that during a food incident, they play two roles. First, they play a role in communicating information to consumers by acting as a conduit for information between the public and the relevant authorities. Second, they play a role as investigators by acting as a public watchdog.

**Conclusion:**

Media actors are an important source of consumer information during food incidents. Public health professionals can work with media by actively approaching them with information about food incidents; promoting to media that as public health professionals, they are best placed to provide the facts about food incidents; and by providing angles for further investigation and directing media to relevant and correct information to inform such investigations. Public health professionals who adapt how they work with media are more likely to influence media to portray messages that fit what they would like the public to know and that are in line with public health recommendations and enable consumers to engage in safe and health promoting behaviours in response to food incidents.

## Background

Previous research has shown that the media play a role in shaping consumer perceptions during a public health crisis [1–6]. Communication through the media is a fundamental component of health promotion strategies that aim to influence consumer health behaviours [7]. The media can influence individuals through: setting the agenda and defining public interest; framing issues through selection and salience; indirectly shaping individual and community attitudes towards risk; and feeding into political debates and decision making [8].

The importance of public health professionals working with the media has previously been reported. It is vital that public health professionals work with the media because ‘if an issue does not exist in the media, then it is not really an issue for decision makers and the larger public’ [9] (p. 299). Additionally, the way in which public health professionals work with media, including how they choose to approach the media about an issue, can either promote consumer health or act as a barrier to improving health status [9]. In particular, forming relationships with media, rather than engaging with them reactively solely when an issue that the public needs to be alerted to arises, will increase media understanding of public health issues [10].

There have been investigations into how media report certain issues including obesity and chronic disease [11–16], dementia [17], swine flu [18], cervical cancer [19], alcohol [20] and long term health conditions [3–5]. There is also some research into journalists’ views about their self-perceived role and approach to reporting public health issues including swine flu [21] and health research [22]. In the context of food and media, investigations have most recently centred around how media frame food risk [23], how food incidents are reported in the media [24], the risk communication strategies used to manage food incidents (including media reporting) [25, 26] and factors influencing the media reporting of food safety issues [27]. Media play an important role in communicating information about food to consumers [23, 28–30]. They are influential in shaping consumer food consumption and attitudes [31] and have a vital role to play in conveying risks related to food [32]. For example, major food incidents across the world have highlighted that media have an important role in informing the public during these incidents and that media reports can affect public trust in the organisations and people portrayed in the stories. However, there has been minimal investigation into the perspectives of media with regards to their role in reporting food incidents. This paper arises from a study investigating the actors that break and reinforce trust in the food system [33]. It reports the perception of media actors’ role in the reporting of food incidents where a food incident is defined as ‘any situation within the food supply chain where there is a risk or potential risk of illness or confirmed illness or injury associated with the consumption of a food or foods’ [34]. Considering the acknowledged importance of the media’s role in influencing public sentiment about food, the lack of information about media perceptions of its own role in the reporting food incidents is surprising.

If public health professionals could communicate their messages about food incidents more effectively through the media, this would contribute to informing the media and consumers with information in line with public health recommendations. However the disconnect between public health professionals and the media – for example the perceived difference in values – is a barrier to this occurring [7]. Therefore this paper aims to contribute to reducing this disconnect by presenting media’s views on their role in presenting information about food incidents and using this information to provide insights for public health professionals about how to work with the media. This paper is important because by seeking to understand media actors’ perspectives about their role, public health professionals can increase their understanding of how to work with media and ultimately portray a message in the media which enables consumers to engage in safe and health promoting behaviour.

## Methods

The study reported in this paper is part of a larger study looking at trust in the food system across three countries. A protocol paper outlining the wider study on which this paper is a part has been published elsewhere [33]. In this paper we use the term ‘media actors’ to refer to individuals currently working within the media, or with previous experience working within the media, including print, radio and online.

### Sample and procedures

Participants were recruited through purposive sampling from Australia, New Zealand (NZ) and the United Kingdom (UK), using two methods. First, industry and network contacts of the research team who were familiar with the work of media actors suggested media actors to speak with, who had demonstrated experience and/or a strong interest in reporting food issues. These people were contacted directly using email. If those approached did not respond to the initial email, a reminder was sent and this was followed up with a phone call. Second, an invitation to participate was sent to media actors including journalists and editors by email through the Communication & Stakeholder Engagement Section at Food Standards Australia New Zealand (FSANZ; project industry partner). The media actors approached through FSANZ also had demonstrated experience in reporting food issues. Therefore, the overall recruitment strategy resulted in recruitment of media actors who had some experience, and/or interest, in reporting food issues. Many also had experience in health and/or science writing. A sampling strategy was devised to ensure coverage of different media formats (online, print (including broadsheet and tabloid), radio and television) and media actors in different positions (journalists, editors, producers, public relations). Project industry partners (TW and DM) were involved in recruitment of participants as outlined above and contributed to study design and data analysis as did other members of the research team, through fortnightly team meetings.

### Measures

#### Interview schedule

The interview schedule (Table 1) was developed based on previous research about food and trust [28, 35, 36] and comment from the research team. It was piloted with two media actors in Australia and two in the UK to check for usability. It was used as a guide during interviews and minor alterations were made as the interviews progressed based on the emergence of new, recurrent themes (for example, the addition of questions about factors influencing media reporting). The interview schedule was designed to discuss media responses to food incidents in general, and in context of a specific, hypothetical scenario (Table 2). The scenario was chosen because it is based on a real scenario and was designed as a safe conversation starter and to give the interviewee a chance to comment on a hypothetical situation as well as relate it to personal experience if desired.

**Table 1:** **Interview schedule used with media actors**

Section of interview	Example questions
Hypothetical scenario	• What would make this story newsworthy?
	• Would you run with this story? Why or why not?
	• What is the immediate story? What are the underlying issues that the media would follow up?
	• What key words would you put in your headline? What angle would you take on the story?
	• What sources would you seek and why?
	• What would you draw on to frame/anchor the story?
	• What risks would you identify in this case that you would seek to convey to consumers?
	• What reaction would your story elicit in consumers?
	• What risks would you identify in this case that you would seek to convey to readers/listeners?
	• What impact do you see your story/reporting having on consumer trust?
General questions	• Please tell me about your role and duties within the media
	• What is the media’s role in contributing to reader/listener trust in food?
	• Do media influence a reader/listener’s decision to trust? Why or why not?
	• What do you see as the media’s role in reporting information to consumers during a food incident?
	• What responsibility do the media have when publishing a story?
	• Do you think that the media seeks to sway public opinion about food incidents in a positive or negative way?

**Table 2:** **Hypothetical scenario used in interviews with media actors**

Scenario	Elements
	• Large food manufacturer has identified contaminated soy protein isolate during routine testing of raw ingredients
	• Source of contaminated soy protein isolate is an Asian country
	• Soy protein isolate is used extensively in the food industry to increase the protein content of a wide variety of foods and drinks that are consumed across all age and social groups
	• Soy protein isolates are also used in infant formulas
	• Subsequent testing has identified the contaminated soy protein isolate in leading brands of infant formula, breakfast cereal, bread and other products that are currently on sale
	• The contaminated product is potentially hepatotoxic, containing a toxin that causes acute liver disease
	• Literature suggests that the toxin can be fatal in vulnerable groups such as children, pregnant women and older people

#### Interviews

Interviews were chosen as the data collection technique to enable open-ended exploration of the topic with participants. Interviews in Australia and New Zealand were conducted by the same researcher. Interviews in the UK were conducted by two researchers. All three interviewers met fortnightly using Skype during data collection to ensure a standard procedure was followed and to reflect on their own influences on the data collection. At these meetings, the three interviewers discussed the data emerging from the interviews and reflected on the ease of discussion. Small changes were discussed and implemented at these meetings, for example minor changes to the interview schedule were made to facilitate ease of the interview based on the interviewer’s reflections. Interviews were conducted either face to face or over the telephone, based on the geographical location and/ or preference of the participant. Interviews in Australia and the UK were conducted between January and March 2013 and NZ interviews were conducted in October 2013.

Interviews were conducted until theoretical saturation of themes was achieved [37]. All interviews (face to face and telephone) were recorded using a digital voice recorder.

### Data analysis

Interview schedules were transcribed verbatim, deidentified and imported into NVivo 10.0 (QSR International, Doncaster). In this study, nonverbal cues, tempo and emphasis were deemed less important and hence were not recorded through the transcription process. Thematic analysis was used to analyse data, using the six phases including familiarising yourself with the data, generating initial codes, searching for themes, reviewing themes, defining and naming themes and producing the paper [38]. Data were coded into themes by one researcher (AW), using a start list of codes that were developed from the research objectives and what was identified as important in the previous research. These included: role of the media in the construction of stories, role of the media in reporting food incidents, media’s perceptions of its role in consumer food trust, use of social media, and sources used. As data were coded, further themes and sub-themes were added based on the objectives of the research. Codes were discussed with other members of the research team who reviewed up to five transcripts each to confirm the themes arising from the primary researcher’s analysis. Data evident in codes was used to develop a framework summarising the primary question of the paper that is how media see their role in reporting of food incidents. Quotes were chosen to include in the paper based on how they demonstrated each area of the framework.

### Ethics approval

This research received ethics approval from the Flinders University Social and Behavioural Research Ethics Committee. Written informed consent was obtained from all participants. This study complies to the RATS guidelines for reporting qualitative studies.

## Results

Details of participants, including the type of media they worked for and their role are reported (Table 3). Eight media actors approached declined to participate (five in Australia and three in the UK).

**Table 3:** **Participant details of media actors including type of media and role (n = 19)**

Country	Type of media	Role	Number of participants	Research codes
Australia	Newspaper	Journalist	3	AUM11, AUM16, AUM15
	Newspaper	Editor	2	AUM6, AUM17
	Television	Journalist	3	AU M1, AUM2, AUM19
	Radio	Radio presenter	3	AUM10, AUM13, AUM18
	Online	Newspaper editor/producer	3	AUM5, AUM7, AUM9
	Online	Blogger/freelance writer	5	AUM3, AUM4, AUM8, AUM12, AUM14
United Kingdom	Newspaper	Journalist	3	UKM5, UKM6, UKM13
	Television	Director/producer	2	UKM10, UKM11
	Radio	Producer	3	UKM1, UKM2, UKM3
	Online	Journalist	1	UKM4
	Varied	Public Relations Consultant	2	UKM14, UKM16
New Zealand	Online	Blogger	1	NZM1
	Newspaper	Journalist	1	NZM2
	**Total**		**33**	

### Media actors’ role in reporting food incidents

**Figure 1:**
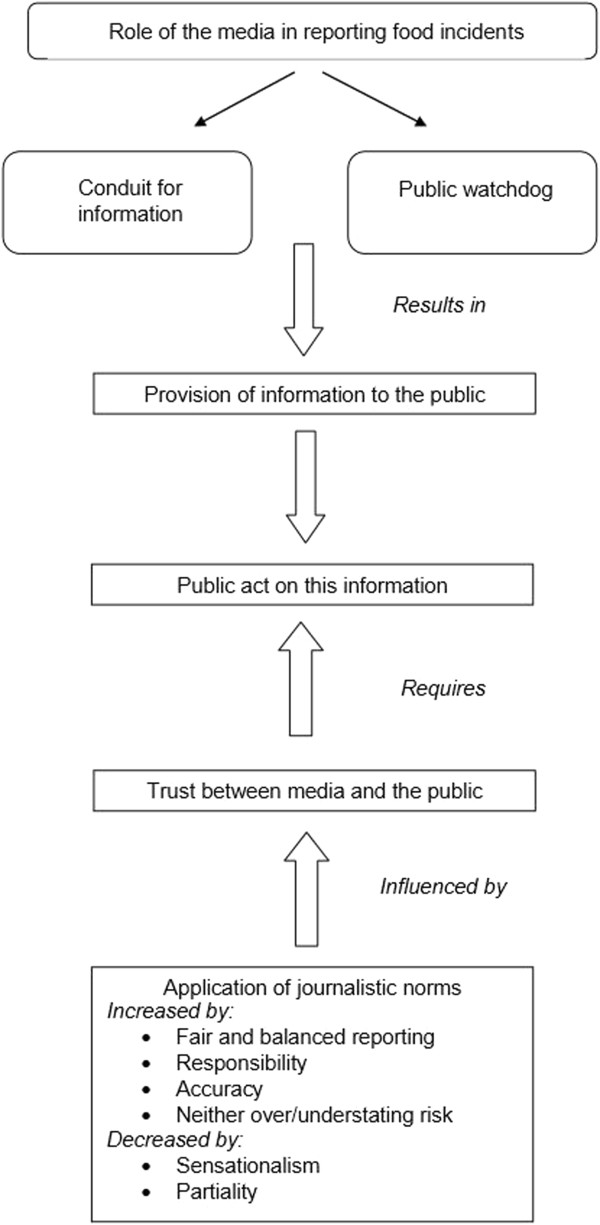
**Media actors’** **role in reporting food incidents.**

Media actors' views on their roles in reporting food incidents are summarised in Figure 1. Participants presented an unanimous view that media have a central and important role in reporting food incidents to the public. This was summarised by one participant who stressed the centrality of the media in reporting food incidents, questioning how food recalls^a^ and health warnings would be communicated to consumers if not disseminated through the media (AU M10^b^).

…*obviously if there*’*s a recall or a health warning*, *if you don*’*t disseminate it through the media*, *then how*’*s it going to get out there*? (*AU M10*) Media actors described this role in reporting food incidents in two main ways: first, as a conduit for information and second, as a public watch dog.

### Media as a conduit for information

Media actors referred to themselves as “*a conduit for information about what*’*s going on*” (AU M15) between consumers and the relevant authorities. One participant described this as follows:

“....*we*’*re really only there as the voice of the public*.... [ ]....*We ask the questions the public would like answered by public health authorities and doctors and that*’*s all we are. We*’*re just the middle men*; *we*’*re just the communicators*”. (*AU M2*)

This role was also described as a community service and public interest role, whereby media actors “*amplify the information released by Government and industry*, *especially regarding* (*food*) *recall information*”. (AU M14)

Some media actors extended the idea of media as a conduit for information by suggesting that part of this role as a conduit is a role in translation of a message provided by other sources, for example Government. One media actor said “*we should report whatever FSANZ* [*Food Standards Australia New Zealand*] *says about the situation*” (*AU M11*). The role of the media in acting as a voice for the public was also described as translating the message for the public and “*making sense of the pronouncements*” made by medical or health science (UK M2). Essentially, the result of media having a conduit role in reporting information was described as enabling the public to decide on a course of action themselves, after the facts had been reported:

*My only agenda is to expose the information*, *let people make the decision after they*’*ve got it*. (*AU M18*)

This view was reiterated by another media actor:

*We*’*re motivated by presenting a balanced story so if the company or the food producer wants to put their point of view of course we*’*ll run that as well and you have to balance that up against whatever the risk is and let the public decide for themselves*. (*AU M2*)

This quote demonstrates a reflection upon the lack of responsibility of the media for the information that is provided to the public. However the need for media to ensure that the information presented to the public is accurate, so that the public can effectively decide on a course of action for themselves, was highlighted.

*We*’*re the go*-*between*, *we*’*re the messenger*, *but we have to be very careful that the message is accurate*, *correct*, *credible*, *responsible and*, *yeah*, *as far as food safety it*’*s very much a case of it coming from the accredited official authorities who do the testing and it*’*s their word that we*’*re conveying*. (*AU M16*)

To manage this, participants cited food safety, Government and health sources as those they would most frequently approach for information during food incidents.

### Media as a public watchdog

Some media actors described an extension of the role of a conduit to a role as a public watch dog, or an investigative role, where media might follow up a food incident story with an investigative lens in order to expose truth and any further issues that do not appear initially in a situation, with a view of ‘watching out’ for the public’s interests. For example:

*Then I think there would be kind of like a deeper investigative role of covering how something like this* [*food incident*] *can happen in the first place*, *how you can prevent it happening and*, *yeah*, *further implications for the food system*, *the global food system*. (*AUM1*)

In doing so, media has a role in investigating suspected issues and holding organisations to account, as described here by one media actor:

*The secondary part would be to*, *I guess*, *continue to investigate the matter and give* – *to balance out whatever the public relations perspective is being distributed by the company itself. The company has obviously* – *they have to provide a certain level of information and I guess our role would be to question that*, *analyse that*, *see if there*’*s actually more to the story than what they*’*re suggesting*.... *our idea is to say* ‘*okay*, *are we saying* – *is this the full*, *360 degree view of this story*?’ *because the company*’*s only going to give you one side*, *which is their side*, *and obviously in a situation like this there*’*s many more people involved that tend* – *it*’*s not the company*’*s job to reveal that but it could be as a media organisation that would be something we would do*. (*AUM6*)

In this example, there is clear distrust of the food company by the media to report factual and complete information.

An investigative role was described as involving questioning and scepticism and exposing what is going on. This was taken to a higher level by some participants who demonstrated a passion for informing the public:

*My role basically is to ensure that people are well aware that there is a risk out there and there are not the safeguards that we would like to minimise that risk or nullify it altogether.* (*AUM18*)

For some media actors, this was the main agenda in being a journalist, as framed by one UK media actor, ‘*a journalist has got to interrogate and investigate*’ (UKM2). A similar view was held by another participant:

*I*’*ve got no agenda other than to try and tell people what*’*s going on and to uncover things that people don*’*t know about.* (*UK M11*)

### Result of media roles on the public’s actions

The result of media actors perceiving their roles in reporting food incidents as conduits for information and public watch dogs was that information about a food incident is provided to the public, with the assumption that the public then act on this information. For example:

*If this product is in your home and it is as potentially harmful as we*’*ve discussed then there*’*s a purpose of making sure that people are aware and have information that they can act on if they have bought these products.* (*UK M3*)

However it is clear from media actors’ reports that whether or not the public act on what the media report is dependent on the extent to which the public trust the media. This reinforces previous reflections by media actors that media actors themselves do not perceive a personal responsibility for consumer’s safety.

### Public trust in the media

Media actors described why it is vital to form trust with the public:

*We*’*re in business to sell papers*, *no*-*one shies away from that*, *however selling papers rests fairly and squarely on building up a long*-*term reputation of credibility*, *of trust*, *of accuracy and that*’*s not something you*’*re going to blow with one story that*’*s going to leave your business struggling for years afterwards to try and rebuild that reputation and rebuild that trust and connection with the broader public who buy the paper*….[ ] …*you can*’*t take that for granted*, *especially in these days of falling circulation*. (*AU M16*)

This was supported by a media actor from New Zealand who talked about the importance of readers trusting what she writes:

*And trust*? *Well I guess I work very hard to make sure what I write is accurate*, *it*’*s scientifically accurate and it*’*s based on current information and that the advice is interpreted correctly and* - *because I want what I write to be something that consumers can trust. I want them to be able to look at my name and say* ‘*if she*’*s written that article then I know it*’*s researched well*’. *So I guess for me the fact that a reader could trust me is very important to me. It*’*s one of the key things*; *it*’*s what I work really hard at*.

Therefore developing trust with the public and a good reputation through the presentation and reporting of accurate, trustworthy information was seen as vital by media actors. Ways in which the public’s trust in media is influenced, both positively and negatively, were discussed.

### Facilitation of public trust

Media actors indicated that trust with the public is facilitated through the application of journalistic norms including responsibility in reporting, accuracy, fair and balanced reporting and not overstating or understating the risks.

Media actors consistently referred to the responsibility they uphold when reporting food incidents:

*I think you*’*d find in most cases the media would react to something like this*[*scenario*] *in a fairly responsible way because* - *as they do in times of any other emergency*, *like bushfires or natural disasters*, *things like that*, *the main thing is to get the story out there*. (*AU M2*)

This notion of responsibility was also demonstrated through a conveyed belief that it is the role of the media to present the facts, be accurate and convey the truth:

*The nature of a food safety issue means it is imperative to report the facts. It*’*s just not the kind of topic that is more or less doing anything but the most objective*, *straightforward reporting on it*. (*AU M6*)

For one participant, accurate reporting by the media during a food incident was assumed:

*I mean obviously they need to report it accurately but I would assume that they do that. I*’*ve no reason to think that the media doesn*’*t report these things accurately*….*For example on the horsemeat* [*scare*], *I*’*m not aware that there was any sort of great* ‘*oh the media got it all wrong and they told us things that weren*’*t correct*’. (*UK M6*)

The idea of balance and treading a careful line between over or understating risk was considered important. However at times this was considered a challenge by participants, with the balance between “*neither overstating nor understating the risks*” (AU M11) needing to be achieved “*in such a way that it doesn*’*t unnecessarily terrify people or create that sort of sensationalism*” (*AU M15*). Similarly a participant from New Zealand said it is ‘*the responsibility of the general media is to present both sides*, *balanced*, *not to frighten people and to have some proportionality* – *you don*’*t want to lull people into false sense of security but you also don*’*t want to scare. You need to inform the public in a way that they can make their own informed choices*’ (*NZM9*).

Participants indicated that it is the role of the media to engage in fair and balanced reporting. When asked about the definition of fair and balanced reporting, responses included “*getting a range of perspectives*” (AU M15), “*as wide a range of views as possible*” (UK M 1) and not putting “*a particular spin on it*” (AU M2). One media actor warned against ‘false balance’ in reporting, which was described as:

*Certain sections of the media have been accused of this thing called false balance* – *if you have a story*, *you would get the opposing views even if it was not a kind of fifty*-*fifty split*, *about*, *for example*, *climate change*, *that you interview somebody about climate change*, *and then you*’*d interview somebody who was opposed*. (*AUM2*)

Inhibition of public trust

On the other hand, media actors said that sensationalism and partiality, which may be enacted by some media actors, did not facilitate trust with the public. One participant, whose role as a media actor involved the use of online media, with strong connections to providing news for a specific industry, indicated that media do not necessarily present fair and balanced information:

*I don*’*t think media generally these days seems to need to pursue balanced and ultimate accuracy in their reports*, *it*’*s all about eyeballs and unfortunately I think that makes it very difficult to achieve the sort of outcomes that the consumer really needs to make balanced judgments about things like a food safety episode*. (*AU M7*)

This view was supported by a media actor who described herself as not impartial:

*I*’*m not in the business of being impartial. I never pretend to be impartial. People come to me for a view and they know the kind of view that I*’*m going to have*. (*UK M6*)

Another participant, who worked in the science sector, agreed with this perspective and even went on to discuss how media will “*look for conflict when there may not be conflict*” in order to sell papers (AU M19). Another believed that fair and balanced reporting may not be the purpose of every section of the newspaper, for example “*if you*’*re writing an opinion piece for a food section or for any other section then obviously it*’*s more subjective and your own opinions are given more credence*” (AU M17) and “*I think as a journalist*, *you don*’*t always have to sit on the fence. I think to have your own opinion is something that is needed but that*’*s very different from a news story in the paper*” (UK M4). However, another, who worked in the higher education sector, identified that while fair and balanced reporting may be ideal, this can be difficult in a newsroom context where “*immediacy really flies in the face of things like balance*, *fairness and so on because there just isn*’*t necessarily that time*, *so while journalists might be more aware of the need for it*, *it doesn*’*t always play out*” (AU M15). Such barriers to balance were reiterated by other media actors who mentioned the time pressure of media reporting and the high turnover of news.

Sensationalism was named by some media actors as being a problem of media reporting, for example ‘*some members of the media seem to go out of their way to look for* “*scare*” *stories*, *to the point*, *in my opinion*, *of irresponsibility*’. (*AUM14*) and:

*I guess that goes back to what I was saying about the constraints of the traditional mainstream format of looking for the most sensational angle so*, *yeah*, *that does lend itself to not necessarily providing balanced*, *useful coverage*. (*AUM12*)

However, others had different views, with one media actor describing sensationalism as ‘*looking for emotive angles*’ (UKM5) and this was further explored by an Australian media actor who said that ‘*the media not only deals with factual information but includes emotional reactions to that information. That is often construed by our critics as* “*sensational reporting*”. (AUM2)

Clearly there were different views about the presence of sensationalist reporting amongst media actors.

## Discussion

This paper presents the views of media actors from three countries about their role in reporting food incidents. The purpose for publishing such a paper in a public health journal is the premise that public health professionals will be more successful in working with the media if they can adapt how they work with media based on their understandings of how media actors understand and perceive their own roles. Media actors see themselves as having two main roles when it comes to reporting information about food incidents – acting as a conduit for information and acting as a public watchdog (Figure 1). There were no clear differences in the understanding of these roles between media actors from different countries or those working across different media formats. The result of media actors enacting these roles is that the public receive information about what to do during food incidents, which they can then act upon. The media actors do not identify responsibility for how the information is received and acted upon. However the data show that there needs to be trust between consumers and the media, and this is influenced by a number of factors which we describe in this paper.

Media actors identify their role as acting in the public interest through providing a conduit for information and acting as a public watchdog (Figure 1). Therefore, clearly the media identify a role in providing the public with knowledge about food incidents. This represents an opportunity for public health professionals to work with media to ensure that the information provided to the media – and consequently presented to the public – is accurate and not misleading, and therefore consumer behaviour during a food incident is appropriate for the situation, safe and health promoting. Of interest is that media actors appeared to make assumptions about the trustworthiness of the source that they received information from, with media actors being less trusting of information provided by the food industry, with the assumption that during a food incident, food companies are ‘guilty until proven innocent’. On the other hand, information about food incidents provided by food regulators was assumed to be correct and not misleading. This represents an opportunity for regulators and other public health professionals as it suggests that there is already a degree of trust in these professionals by the media. It has been identified in other studies that media seek to broadcast the opinions of public health officials [21].

The media have been identified as an important source of health information for the general public [39], and this study confirms that media actors see themselves as having a role in communicating information about food incidents. This is confirmed by other studies. In one study, ten percent of the population reported using the mass media as their main source of information about health related issues [40]. The media operates ‘as an interface between the medical community, government and the public [and] it therefore plays a critical role in shaping public opinion regarding health issues’ [41], or as identified in this study, acting as a conduit for information by providing the information to consumers who then make their own decisions. In this study, media actors also view their role as acting as a public watchdog through provision of credible information and balanced reporting but also though investigating claims to arrive at the ‘truth’. This highlights that media personnel are concerned not only about presenting the facts through their role as a conduit for information but also concurrently checking that information through their role as a public watchdog, which can be described as upholding a responsibility to check the information presented to consumers. A study of media personnel reporting on the swine flu epidemic found that journalists ‘articulated a clear commitment to their roles as journalists: as public informants, independent and neutral’ [21]. Likewise, Forsyth et al. [22] found that journalists saw their ‘primary responsibility to be the education of the lay public’ and in the context of their reporting, expressed commitment to journalistic principles including accuracy, balance and independence. The presentation of information and exposure of misconduct which is obtained through investigation, or a public watchdog role, are both viewed as important aspects of journalistic work.

Despite the fact that media actors reported they uphold journalistic norms such as fair and balanced reporting and accuracy, for example, it must be questioned whether this is actually the case. Some of the media actors in this study did say that the media do put a particular slant on information when it is reported and engage in sensationalist reporting. Interestingly, those media actors who had worked outside media (for example higher education, science and industry sectors) were more likely to acknowledge external factors that may compromise fair and balanced reporting. Other participants did, however, identify workplace factors such as immediacy and newsworthiness which impact capacity to provide fair and balanced reporting. For journalists working in newsrooms, editors act as gatekeepers to what is presented. Cross media ownership and presentation of information on multimedia sites has eroded the autonomy of the individual journalist [42].

Örnebring [43] argues that these changes have been associated with a reconstruction of the concept of autonomy for those working in traditional media leading to a focus upon institutional rather than individual autonomy. What is valued is editorial autonomy and the reputation of the media which is associated with the trustworthiness of the medium. This is reflected in this study in the claim that the reputation of, and trust in the media source relates to the credibility and truthfulness of the information provided. Truth is understood differently via media however. For traditional media truth telling is associated with the presentation of accurate information while for bloggers it is associated with the presentation of a variety of views which enable the reader to arrive at their own conclusion [44]. Truth telling in turn, is related to public trust in the media as a source of information on which to base informed choice [44]. Our participants describe their role in terms of serving the public through providing timely and objective information and through investigating food issues to arrive at a truth.

It has previously been discussed that journalistic norms are ideological whereby the media position themselves as having these norms in order to assist in protecting professional boundaries and presenting journalism as a legitimate profession [45]. Deuze [42] argues for the development of a professional ideology in journalism in the 20th century based on shared values that “validate and give meaning” to journalistic work. The professional ideology for Deuze [42], consists of five ideal- typical traits including: provision of a public service through acting as a public watchdog; objectivity; professional autonomy; immediacy through presenting information as it becomes available; and journalistic ethics most commonly expressed as truth telling. For Deuze [42] these values legitimate journalistic practice. Lewis questioned whether these standards are truly reflected in practice or whether they are used to justify practice and to create professional boundary in a profession which lacks the characteristics of a traditional ‘profession’ [45]. This study supports both views with the majority of actors saying that they uphold journalistic norms but others, who sit outside media, saying that they are used to justify practice. However what is important is that the views portrayed in this paper indicate how different media actors see themselves and their work, and portray themselves to the wider public. These views, while conflicting, can be used by public health professionals to better understand how to work with media.

A strength of this study is the inclusion of media actors from various types of media including television, radio, online and newspaper from three countries. A potential limitation is the possibility that the methods used in this study (interviewing journalists without any prior development of a relationship or trust) would not have elicited views from journalists that indicated they were not acting in the public good (for example actions driven by business values).

It has previously been argued that social scientists need to understand and adapt to the conditions under which media reporting operates if they are to be succeed in introducing the findings of social research into public debates [46]. It would appear that the media can be viewed as a site for conflicting interests [47], where scientific sources manage media content to present themselves in the best light while journalists manage their sources to get the information they want [48]. In this study, media actors suggested that public health sources were more trustworthy than food industry sources. Furthermore, the media tends to reproduce mainstream views of health issues, marginalising views that contradict taken-for-granted understandings of the issue [49]. This creates a challenge for public health researchers who wish to influence public opinion via the media as the frames operating with public health are often at odds with those in the media and information presented may not be in line with public health recommendations. As a consequence, public health researchers may need to frame their findings to attract media attention through highlighting topical or newsworthy content [49] and/ or use their understanding of the positions from which media work, as explored in this paper. This has important implications for public health professionals. If public health professionals and other health practitioners understand how media perceive their role then the practitioners can respond with this in mind through presentation of a high quality message that fits with a public health agenda encouraging consumers to respond to a food incident appropriately.

Science reporters have also been found to use established networks or scientists with a public profile regardless of their expertise for comment on issues [50]. This may require a more proactive approach by public health professionals who can respond to media actors’ perception of themselves as conduits for information through providing the information they wish to be communicated to the public, rather than waiting for the media to find information from another source. Furthermore, public health professionals can highlight their qualifications and credentials to media actors to demonstrate that they are qualified to communicate the facts of the incident to media actors, who then report the facts to the public. Considering the trust media actors in this research has in public health professionals, demonstrating credibility as a source is not likely to be overly challenging. Finally, public health professionals can work with media as public watchdogs by suggesting areas of interest for investigation and either providing some information that will aid with this investigation and/ or providing direction to where they could find that information. Therefore public health professionals are in a position of power and have the ability to affect the health of consumers through how they choose to engage with the media. Despite this, working with the media is an area where most public health professionals have not been trained [10] and therefore this represents an area for development.

## Conclusion

This paper adds to debate through presenting the views of media actors themselves about their role in reporting food information during food incidents. As indicated in this paper, media represent a crucial avenue through which consumers receive information during food incidents. The media therefore play a vital role in disseminating public health messages. The insights provided in this paper into media actors’ perceptions of their role in reporting public health information in the context of food incidents, might help public health professionals to work better with media, become more media savvy and ultimately ensure that an appropriate consumer response to food incidents which maintains safe and health promoting behavior is upheld. Future research could focus more on cross-country differences in ideas between media actors. It could also seek to better understand the nuances in understanding of roles between media actors from different media formats, and suggest communication strategies specific to media and public health professionals based on this.

## Endnotes

^a^A food recall is an action taken to remove food that may pose a health and safety risk to consumers from distribution, sale and consumption Food Standards Australia and New Zealand (2008). Food Industry Recall Protocol: A guide to conducting a food recall and writing a food recall plan Canberra, Food Standards Australia and New Zealand.

^b^Interview identification system with ‘AU’ indicating Australian interviewee, ‘UK’ indicating UK interviewee, ‘NZ’ indicating NZ interviewee and ‘M’ representing ‘media actor’.
